# NLRP3 Inflammasome in Neurological Diseases, from Functions to Therapies

**DOI:** 10.3389/fncel.2017.00063

**Published:** 2017-03-09

**Authors:** Limin Song, Lei Pei, Shanglong Yao, Yan Wu, You Shang

**Affiliations:** ^1^Department of Anesthesiology, Institute of Anesthesiology and Critical Care Medicine, Union Hospital, Tongji Medical College, Huazhong University of Science and TechnologyWuhan, China; ^2^Department of Neurobiology, School of Basic Medicine, Tongji Medical College, Huazhong University of Science and TechnologyWuhan, China; ^3^Department of Neurology, Union Hospital, Tongji Medical College, Huazhong University of Science and TechnologyWuhan, China; ^4^Department of Critical Care Medicine, Institute of Anesthesiology and Critical Care Medicine, Union Hospital, Tongji Medical College, Huazhong University of Science and TechnologyWuhan, China

**Keywords:** neuroinflammation, NLRP3, inflammasome, microglia, astrocytes, IL-1β, IL-18

## Abstract

Neuroinflammation has been identified as a causative factor of multiple neurological diseases. The nucleotide-binding oligomerization domain-, leucine-rich repeat- and pyrin domain-containing 3 (NLRP3) inflammasome, a subcellular multiprotein complex that is abundantly expressed in the central nervous system (CNS), can sense and be activated by a wide range of exogenous and endogenous stimuli such as microbes, aggregated and misfolded proteins, and adenosine triphosphate, which results in activation of caspase-1. Activated caspase-1 subsequently leads to the processing of interleukin-1β (IL-1β) and interleukin-18 (IL-18) pro-inflammatory cytokines and mediates rapid cell death. IL-1β and IL-18 drive inflammatory responses through diverse downstream signaling pathways, leading to neuronal damage. Thus, the NLRP3 inflammasome is considered a key contributor to the development of neuroinflammation. In this review article, we briefly discuss the structure and activation the NLRP3 inflammasome and address the involvement of the NLRP3 inflammasome in several neurological disorders, such as brain infection, acute brain injury and neurodegenerative diseases. In addition, we review a series of promising therapeutic approaches that target the NLRP3 inflammasome signaling including anti-IL-1 therapy, small molecule NLRP3 inhibitors and other compounds, however, these approaches are still experimental in neurological diseases. At present, it is plausible to generate cell-specific conditional NLRP3 knockout (KO) mice via the Cre system to investigate the role of the NLRP3 inflammasome, which may be instrumental in the development of novel pharmacologic investigations for neuroinflammation-associated diseases.

## Introduction

Neuroinflammation is a fundamental innate immune response in the central nervous system (CNS) by which the brain and spinal cord react to diverse pathogens and host-derived signals of cellular damage. Inflammatory responses are necessary steps for eliminating invading agents, clearing damaged cells and promoting tissue repair (Miwa et al., 1997; Tahara et al., 2006; Ito et al., 2007; Ribes et al., 2009; Szretter et al., 2009); however, uncontrolled neuroinflammation may lead to further tissue injury and neural dysfunction (Lazovic et al., 2005; Choi et al., 2009; Abo-Ouf et al., 2013). Therefore, it has become evident that neuroinflammation represents a significant cause of neurological deficits.

Researchers have recently focused their attention on a group of subcellular multiprotein complexes referred to as inflammasomes (Martinon et al., 2002), whose formation and activation may be induced by a wide range of substances (Kanneganti et al., 2006b; Mariathasan et al., 2006; Martinon et al., 2006; Sutterwala et al., 2007; Newman et al., 2010; Rathinam et al., 2010). In particular, the nucleotide-binding oligomerization domain-, leucine-rich repeat- and pyrin domain-containing 3 (NLRP3) inflammasome has gained considerable attention (Agostini et al., 2004). It is abundantly expressed in the CNS and may serve to detect noxious agents or irregularities in the cellular microenvironment (Halle et al., 2008; Yin et al., 2009; Jha et al., 2010; Geldhoff et al., 2013b; Yang et al., 2014). Activated NLRP3 inflammasome leads to the activation of caspase-1, which mediates the production of interleukin-1β (IL-1β) and interleukin-18 (IL-18) pro-inflammatory cytokines and the initiation of a rapid form of cell death termed pyroptosis (Martinon et al., 2002; Kanneganti et al., 2006a; Fink et al., 2008). IL-1β and IL-18, in turn, initiate multiple signaling pathways and drive inflammatory responses, which results in neuronal injury or death (Yatsiv et al., 2002; Bossù et al., 2010; Meissner et al., 2010; Wilms et al., 2010). Therefore, the NLRP3 inflammasome plays a crucial role in the development of inflammatory responses in the CNS. Moreover, emerging studies have revealed the involvement of NLRP3 signaling in several neurological disorders (Gris et al., 2010; Hoegen et al., 2011; Fann et al., 2013b; Heneka et al., 2013; Johann et al., 2015).

Herein, we describe the general principles involved in the structure and activation mechanisms of the NLRP3 inflammasome in the CNS, as well as the complex neuroinflammatory signaling pathways and consequences associated with NLRP3 inflammasome activation under the circumstances of brain infection, acute injury and neurodegenerative disorders. Finally, we conclude that NLRP3 inflammasome signaling may represent a promising therapeutic target for the treatment of neuroinflammation-associated neurological diseases.

## Microglia and Astrocytes in Neuroinflammation

Emerging evidence supports the notion that various pathological changes within the CNS elicit a prominent inflammatory reaction referred to as neuroinflammation. In the CNS parenchyma, microglia and astrocytes are the primary effectors of neuroinflammation (Karve et al., 2016; Shrivastava et al., 2017). Plasma membrane pattern recognition receptors (PRRs) expressed on glial cells play an important role in the activation of nuclear factor-κB (NF-κB) and mitogen-activated protein kinase inflammatory pathways (Tang et al., 2013; Heneka et al., 2014). In addition, microglial-astrocyte communication is highly important in CNS innate immunity.

Microglia, the resident macrophage-like cells of the CNS, are derived from yolk-sac myeloid progenitors during the early stage of embryonic development (Kierdorf et al., 2013). They continually monitor and survey their assigned brain regions and participate in CNS development, neuroprotection, and the maintenance of hemostasis (Paolicelli et al., 2012; Nayak et al., 2014). Microglia express numerous PRRs that are responsible for the early recognition of pathogen-associated molecular patterns (PAMPs) and damage-associated molecular patterns (DAMPs), such as Toll-like receptors (TLRs), NOD-like receptors (NLRs), retinoic acid-inducible gene-I-like receptors and triggering receptor expression on myeloid cells 2 (TREM2; Shah et al., 2008; Kumar et al., 2011; Hu et al., 2012; Fu et al., 2014). Microglial cells are considered the earliest responders to pathological insults on the CNS (Becher et al., 2000; Saijo et al., 2013). Under non-pathological conditions, microglial cells have a highly ramified appearance. Upon detecting environmental challenges, such as brain injury, infection, or protein aggregates, microglia become rapidly “activated” and transform to an ameboid appearance with increased expression of major histocompatibility complex molecules and other markers (Rock et al., 2004; Kettenmann et al., 2011). Proliferation and migration of microglial cells also occur (Byrnes and Faden, 2007). Moderate microglial responses exert protective effects on the CNS in some circumstances (Lalancette-Hébert et al., 2007). Nevertheless, over-activated microglia can lead to neuroinflammation, oxidative stress and neuronal dysfunction due to the excess production of a wide range of cytotoxic factors, such as tumor necrosis factor-α (TNF-α), IL-1β, IL-18, IL-6, reactive oxygen species (ROS) and nitric oxide (NO; Moss and Bates, 2001; Heneka and O’Banion, 2007; Nayak et al., 2014). Moreover, the interaction between microglia and other immune cells results in secondary inflammatory responses. Recent findings have involved microglia activation in the initiation and maintenance of inflammatory responses in the context of infectious brain diseases, acute CNS injury and several neurodegenerative diseases (Aoki et al., 2009; Fellner et al., 2013; Elmore et al., 2014; Liu et al., 2015; Kumar et al., 2016; Li D. et al., 2016; Xian et al., 2016).

Similar to peripheral macrophages, several broad classifications of microglia M1, M2a, M2b and M2c have been identified in the literature (Geissmann et al., 2010; Franco and Fernández-Suárez, 2015). “Classically activated” M1 microglia, which exhibit an ameboid appearance, strong phagocytic capability and high mobility, are characterized by the generation and secretion of large amounts of pro-inflammatory mediators and high levels of oxidative production (Loane and Byrnes, 2010; Zhang et al., 2016). Generally, “alternatively activated” M2 microglia exhibit a hyper-ramification appearance (Cherry et al., 2014) and produce plenty of anti-inflammatory cytokines, neurotrophic factors, and extracellular matrix molecules (David and Kroner, 2011; Michell-Robinson et al., 2015). Therefore, M2 microglia are primarily associated with the resolution of inflammation and tissue repair. Three subclasses of M2 microglia exhibit slight differences in cellular markers and functions. M2b microglia are characterized by large amounts of pro-inflammatory cytokine generation and by high IL-10 and low IL-12 expression (Ferrante et al., 2013; Orihuela et al., 2016), which allows them to be able to shift to a mixture of M1 and M2a/b.

Substantial numbers of astrocytes are present in the CNS parenchyma, and they perform a diverse array of functions including glutamate uptake, fuel provision, synaptogenesis, structural support and immune defense (Brown and Ransom, 2007; Perea et al., 2009, 2014; Barreto et al., 2011). Astrocytes display a great degree of heterogeneity in their morphology, lineage, anatomical locations and gene expression profile (Zhang and Barres, 2010; Sosunov et al., 2014). Astrocytes are equipped with various innate immune receptors, and they directly respond to different types of CNS insults with hypertrophy and hyperplasia, a process referred to as astrogliosis. Astrocytes, however, are involved in the formation of compact astrocytic scars by forming and orienting long processes toward the core of severely damaged sites (Cregg et al., 2014). Under normal circumstances, reactive astrogliosis and scar formation are essential for confining CNS inflammation to the lesion epicenter, protecting neural networks and promoting repair of the blood–brain barrier (BBB; Pekny and Nilsson, 2005; Sofroniew, 2005, 2009). However, under pathological conditions, astrogliosis can interfere with neurite growth and regeneration (Silver and Miller, 2004; Anderson et al., 2014; Cregg et al., 2014). Moreover, reactive astrocytes produce numerous pro-inflammatory mediators, such as cytokines, chemokines and NO, which makes them essential for exacerbating inflammatory responses. Notably, by regulating specific signaling events, astrogliosis can exert either potent pro-inflammatory effects or essential anti-inflammatory effects.

## NLRP3 Inflammasome

The NLRP3 inflammasome was first characterized in Muckle-Wells Autoinflammatory Disorder (Martinon et al., 2002; Agostini et al., 2004). NLRP3 inflammasome can sense various stimuli and form a molecular platform for caspase-1 activation, which leads to the processing and release of IL-1β and IL-18 and eventually potentiates inflammatory responses that are involved in multiple infectious, inflammatory and immune diseases (Sutterwala et al., 2006; Willingham et al., 2007; Halle et al., 2008; Masters et al., 2010; Walsh et al., 2014). Thus, the NLRP3 inflammasome is of crucial importance in the development of both acute and chronic inflammatory responses.

**Figure 1:**
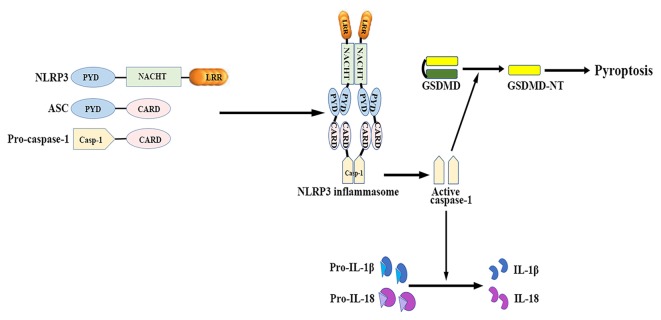
**NLRP3 inflammasome: structure and function.** The NLRP3 inflammasome mainly consists of the cytosolic sensor molecule NLRP3, the adaptor protein ASC, and the effector molecule pro-caspase-1. The assembly and activation of NLRP3 inflammasome results in caspase-1 activation. Activated caspase-1 subsequently leads to the maturation of IL-1β and IL-18, as well as mediates a form of inherent inflammatory cell death termed as pyroptosis. ASC, apoptosis-related speck-like protein containing a caspase recruitment domain; CARD, caspase activation and recruitment domain; GSDMD, gasdermin D; GSDMD-NT, gasdermin-N domain of GSDMD; IL, interleukin; LRR, leucine-rich repeat; NACHT (NOD), nucleotide binding and oligomerization domain; NLRP3, nucleotide-binding oligomerization domain-, leucine-rich repeat- and pyrin domain-containing 3; PYD, pyrin-only domain.

The NLRP3 inflammasome mainly consists of a cytosolic sensor molecule NLRP3, an adaptor protein apoptosis-associated speck-like protein containing a caspase activating recruitment domain (ASC), and a cysteine protease pro-caspase-1 as the effector molecule (Agostini et al., 2004; see Figure 1).

NLRP3 contains a C-terminal leucine-rich repeat (LRR) domain, a conserved central nucleotide binding and oligomerization domain (NOD or NACHT), and an N-terminal pyrin-only domain (PYD; Anderson et al., 2004). The LRR domain recognizes PAMPs and other ligands, maintains the NLRP inactive state, and mediates protein-protein interactions (Meng et al., 2003; O’Connor et al., 2003; Hoffman et al., 2010). The NACHT domain, with ATPase activity, is essential for protein self-oligomerization during the inflammasome assembly process (Duncan et al., 2007). The PYD enables protein-protein homotypic interactions between NLRP and the bipartite adapter ASC (Liepinsh et al., 2003).

ASC consists of an N-terminal PYD and a C-terminal caspase activation and recruitment domain (CARD; Masumoto et al., 1999). ASC binds to the upstream NLRP3 through a homotypic PYD-PYD domain interaction, which results in ASC dimer assembly into a large speck-like structure (Dowds et al., 2004). ASC interacts with pro-casapase-1 via the CARD domain (Srinivasula et al., 2002).

Caspase-1 is present as a catalytically inactive precursor pro-caspase-1 in unstimulated cells. Pro-caspase-1 recruitment, which is mediated by ASC, contributes to caspase-1 oligomerization and auto-proteolytic conversion of the pro-enzyme into its active form (Agostini et al., 2004). The active caspase-1 fragments elicit the maturation and secretion of pro-inflammatory cytokines IL-1β and IL-18, which belong to the IL-1β family and mediate subsequent immune responses (Keller et al., 2008).

Caspase-1 activation also induces a form of inherent inflammatory cell death, referred to as pyroptosis, which is characterized by rapid plasma-membrane rupture, DNA fragmentation, and the release of pro-inflammatory cytosolic contents into the extracellular space (Bergsbaken et al., 2009). Pyroptosis is both morphologically and mechanistically different from apoptosis and other forms of cell death. Recent studies have identified that activated caspase-1 cleaves gasdermin D (GSDMD) to generate the gasdermin-N domain of GSDMD (GSDMD-NT), which can directly bind phosphoinositides and cardiolipin (Shi J. et al., 2015; Ding et al., 2016; Liu et al., 2016). GSDMD-NT then associates with the plasma membrane and oligomerizes to form non-selective pores, which triggers cell swelling and lysis (Chen et al., 2016; Liu et al., 2016). Both neurons and glial cells may trigger this process of cell death in response to a wide range of pathological stimuli (Adamczak et al., 2014; Tan et al., 2014; Kim et al., 2015).

## Activation and Regulation of the NLRP3 Inflammasome

The NLRP3 inflammasome is the most extensively investigated inflammasome, and it is present in microglia and astrocytes in the CNS (Cho et al., 2014; Lu et al., 2014; Zendedel et al., 2016). It remains debated whether neurons express NLRP3 (Fann et al., 2013a; Yang et al., 2014; Kaushal et al., 2015). *In vitro* studies suggest that the basal level of NLRP3 in resting cells is not sufficient to activate the inflammasome. It is widely accepted that successful NLRP3 inflammasome activation requires a two-checkpoint signal process. A priming signal is provided by the NF-κB-activating stimuli to transcriptionally enhance the expression of NLRP3 and pro-IL-1β (Bauernfeind et al., 2009). Many TLR and NLR ligands, as well as endogenous cytokines such as IL-1α, have been demonstrated to prime cells. The subsequent activating signal is provided by various NLRP3-activating agents to promote the formation of the inflammasome complex. A wide range of exogenous and endogenous stimuli including PAMPs, aggregated and misfolded proteins, ATP and crystalline substances induce NLRP3 activation (Mariathasan et al., 2006; Martinon et al., 2006; Halle et al., 2008; Demento et al., 2009; Duncan et al., 2009; Shi F. et al., 2012).

Given the broad array of NLRP3 activators, NLRP3 appears to sense the disturbance of cellular homeostasis rather than directly react to these stimuli. To elucidate this, researchers have proposed several theories as follows: (1) low intracellular K^+^ concentration may play a major role in common signal transduction for NLRP3 activation (Pétrilli et al., 2007; Marina-García et al., 2008; Karmakar et al., 2016); (2) endo-lysosomal destabilization induces the release of cathepsins into the cytosol, which may directly activate NLRP3 (Hornung et al., 2008; Sharp et al., 2009; Bruchard et al., 2013); (3) ROS, mitochondrial DNA and phospholipid cardiolipin released from damaged mitochondria activate NLRP3 (Zhou et al., 2010, 2011; Subramanian et al., 2013); (4) Ca^2+^ flux and the Ca^2+^-dependent signaling trigger the assembly of NLRP3 inflammasome (Feske et al., 2012; Lee et al., 2012; Murakami et al., 2012).

The activity of NLRP3 is finely regulated through distinct mechanisms. Recent studies have revealed that BRCC-3, double-stranded RNA-dependent protein kinase, death-associated protein kinase 1 and Bruton’s tyrosine kinase function as endogenous positive regulators of NLRP3 inflammasome activity (Chuang et al., 2011; Juliana et al., 2012; Lu et al., 2012; Py et al., 2013; Ito et al., 2015). A member of the NIMA-related kinase (NEK) family, NEK7 has been shown to directly bind to the LRR domain of NLRP3 and act downstream of K^+^ efflux and ROS generation to promote the assembly of NLRP3 inflammasome (He et al., 2016; Schmid-Burgk et al., 2016; Shi et al., 2016). Nevertheless, autophagy, microRNAs, CARD-only proteins, pyrin-only proteins and NO act as endogenous negative regulators of NLRP3 (Saitoh et al., 2008; Hernandez-Cuellar et al., 2012; Shi C. S. et al., 2012; Mishra et al., 2013; de Almeida et al., 2015; Qin et al., 2015; Yang et al., 2015).

## Effects of Inflammasome Activation on Neuroinflammation

**Figure 2:**
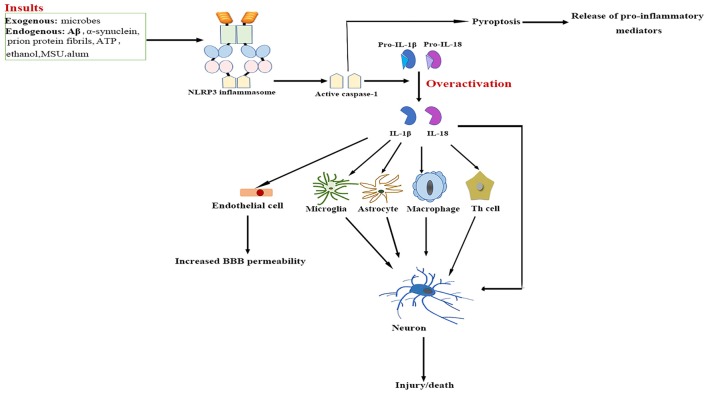
**NLRP3 inflammasome activation-mediated neuroinflammation.** Upon activation by a wide range of exogenous and endogenous stimuli, the NLRP3 inflammasome located in microglia and astrocytes trigger the maturation of IL-1β and IL-18 and induce pyroptotic cell death. The high levels IL-1β and IL-18 bind to their receptors on glial cells, neurons, macrophages and endothelial cells, as well as cooperate with other cytokines to initiate Th-cell signaling, thereby triggering a complex spectrum of signaling events, which results in exacerbation of inflammatory cascade responses within the central nervous system (CNS). Aβ, β-amyloid; ATP, adenosine triphosphate; BBB, blood–brain barrier; IL, interleukin; MSU, monosodium urate; NF-κB, nuclear factor-κB; NLRP3, nucleotide-binding oligomerization domain-, leucine-rich repeat- and pyrin domain-containing 3; Th, T helper.

The NLRP3/caspase-1/IL-1 axis has emerged as a critical signaling pathway of the innate immune system in the CNS (Rosenzweig et al., 2011; see Figure 2). The abundance of caspase-1 has been identified in the context of neuroinflammation-related disorders (Sifringer et al., 2007; de Rivero Vaccari et al., 2016). IL-1β and IL-18 are cytokines that are matured by the NLRP3 inflammasome. The involvement of IL-1β and IL-18 in neuroinflammation has long been speculated (Arend et al., 2008; Dinarello et al., 2012). High levels of IL-1β and IL-18 have been demonstrated in the cerebrospinal fluid (CSF), brain tissue and plasma of patients with CNS infection, brain injury and neurodegenerative diseases such as Alzheimer’s disease (AD) and multiple sclerosis (MS; Licastro et al., 2000; de Jong et al., 2002; Huang et al., 2004). Both IL-1β and IL-18 bind to their respective receptors on microglial cells, astrocytes, neurons and endothelial cells, thereby triggering a complex spectrum of signaling events, which results in secondary expression of multiple inflammation-associated genes. Notably, cytokine-mediated processes were shown to be involved in cognitive decline and have been verified to lead to long-term neuropsychiatric disorders (McAfoose and Baune, 2009).

IL-1β signaling plays a major role in the initiation and continuation of the inflammatory reactions in the CNS in response to various adverse stimuli (Cannon, 2000). IL-1β contributes to modulating the integrity of the BBB, which results in the infiltration of peripheral immune cells into the CNS (Alvarez et al., 2011). IL-1β also stimulates the activation of microglia and astrocytes, which in turn activates CNS-infiltrated T cells and induces the generation of additional pro-inflammatory factors such as IL-6 and TNF-α, as well as neurotoxic mediators (Ferrari et al., 2004). Moreover, IL-1β indirectly recruits leukocytes by augmenting the expression of chemokines (Gosselin and Rivest, 2007). Several experimental studies have even demonstrated that over-expression of IL-1β mediates neuronal injury by regulating glutamate excitotoxicity (Hu et al., 2000; Fogal et al., 2007).

IL-18 mainly stimulates T-helper (Th) cell-mediated immune responses by inducing the production of adhesion molecules, pro-inflammatory cytokines and chemokines in natural killer, Th1 and B cells (Nakahira et al., 2002; Bossù et al., 2010). IL-18 also activates signaling pathways in microglia, which results in increased caspase-1 expression and matrix metalloproteinases and pro-inflammatory cytokine production (Felderhoff-Mueser et al., 2005). In addition, IL-18 augments Fas ligand expression in glial cells, thereby exacerbating Fas-mediated neuronal cell death in the context of neuroinflammation. IL-18 thus converges two distinct immunological regulatory pathways of inflammatory reactions and cytotoxic effects.

Pyroptosis is a highly inflammatory, programmed form of cell death. It is distinct from necrosis and apoptosis as the pathway is exclusively mediated by activated caspase-1 (Bergsbaken et al., 2009). So far, pyroptosis has been described in both glial cells and neurons (Alfonso-Loeches et al., 2014; Tan et al., 2014; Kim et al., 2015). Pyroptosis causes rapid rupture of the plasma membrane and excessive release of pro-inflammatory cytokines and chemokines such as TNF-α, IL-1β, IL-6 and CX_3_C-chemokine ligand 3, which may aggravate inflammatory mediator-induced neuronal death (Fink et al., 2008; Ramesh et al., 2013). As these factors have been shown to mediate the recruitment of other immune cells from peripheral circulation, plenty of leukocytes are attracted to the inflammation sites, and the subsequent inflammatory responses cause severe tissue damage in the CNS under neuropathological conditions (Koedel et al., 2009).

## NLRP3 Inflammasome and Neurological Diseases

Aberrant activation of NLRP3 inflammasome signaling has been demonstrated to contribute to pathology in a broad spectrum of neurological diseases (see Table 1).

**Table 1:** **Neurological disorders that involve the NLRP3 inflammasome**.

	Disease	Current animal models	Reference
Brain infection	*S. pneumoniae* meningitis	Intracisternal inoculation of *S. pneumoniae* strain (mouse)	Hoegen et al. (2011), Geldhoff et al. (2013a), Kim et al. (2015)
	Japanese encephalitis	Intravenous injection of Japanese Encephalitis virus (mouse)	Kaushik et al. (2012)
	Influenza virus infection	Intranasal infection of influenza virus (mouse)	Yu et al. (2014)
	HIV/AIDS	Feline immunodeficiency virus infection (cat); HIV-1 Vpr transgenic mouse	Mamik et al. (2016)
Acute injury	Cerebral ischemia	Focal cerebral ischemia: transient middle cerebral artery occlusion (mouse, rat); global cerebral ischemia: bilateral 4-vessel occlusion (rat)	Fann et al. (2013b), Yang et al. (2014), Wang et al. (2015), Thakkar et al. (2016)
	Traumatic brain injury	Modified Feeney model (mouse); controlled cortical impact (rat); Blast-induced traumatic brain injury (rat)	Liu et al. (2013), Ma et al. (2016), Lin et al. (2017)
	Spinal cord injury	Dorsal root avulsion (rat); spinal cord contusion lesion (rats)	Ellis et al. (2016), Jiang et al. (2016), Zendedel et al. (2016)
	Subarachnoid hemorrhage	Endovascular perforation model (rat)	Li et al. (2016), Shao et al. (2016)
	Intracerebral hemorrhage	Autologous blood injection (mouse)	Ma et al. (2014), Yang et al. (2015)
Neurodegenerative diseases	Alzheimer’s disease	APP/PS1 mouse; TgCRND8 AD mouse; 3xTgAD mouse; stereotaxic injection of β-amyloid	Heneka et al. (2013); Cho et al. (2014); Liu et al. (2015), Daniels et al. (2016), Dempsey et al. (2017)
	Multiple sclerosis	Experimental autoimmune encephalitis	Jha et al. (2010), Inoue et al. (2012), Coll et al. (2015)
	Amyotrophic lateral sclerosis	SOD1(G93A) mouse model	Johann et al. (2015), Debye et al. (2016)
	Prion diseases (remains controversial)	Prion inoculation (mouse)	Nuvolone et al. (2015)

### Brain Infection

#### Bacterial infection

*Streptococcus pneumoniae (S. pneumoniae)* causes meningitis when it invades the CSF space. Studies of both murine models and patients have demonstrated that the NLRP3 inflammasome plays a central role in the pathologic progression of pneumococcal meningitis (Hoegen et al., 2011; Geldhoff et al., 2013a). Pneumolysin, a pneumococcal pore-forming cytolysin, induced caspase-1-dependent pyroptotic cell death and IL-1β maturation through ATP-dependent lysosomal destabilization and ROS production (Kim et al., 2015). Excessive NLRP3 inflammasome activation led to extensive inflammatory responses and exacerbated tissue damage in the brain, as well as other adverse outcomes (Wu et al., 2010; Hoegen et al., 2011; Mitchell et al., 2012). *Staphylococcus aureus* (*S. aureus)* also induced NLRP3 inflammasome activation in microglia in an ATP- and cathepsin B-dependent manner (Hanamsagar et al., 2011). Moreover, priming microglia with conditioned media from Mycobacterium tuberculosis (Mtb)-infected macrophages, in combination with infection with Mtb, instigated robust activation of the NLRP3 inflammasome. Lowering intracellular K^+^ concentrations, lysosomal protease release and mitochondrial ROS generation were suggested to be upstream events of Mtb-induced NLRP3 activation (Lee et al., 2013). Listeria monocytogenes (LM) is the causative agent of several life-threatening diseases, including meningitis and septicemia (Roed et al., 2012; Thønnings et al., 2016). As NLRP3 is one of the major sensors of LM (Warren et al., 2008), it is plausible that NLRP3 inflammasome play a role in the pathology of LM-associated meningitis.

#### Viral Infection

Japanese Encephalitis virus (JEV) represents a common cause of acute viral encephalitis. Microglia rapidly respond to JEV infection and secrete several pro- and anti-inflammatory cytokines, including IL-1β and IL-18. JEV triggers NLRP3 inflammasome activation through K^+^ efflux and ROS production, as shown in a murine model and Bv-2 microglial cells (Kaushik et al., 2012). It has been implicated that NLRP3 inflammasome signaling plays a crucial role in host protection during influenza virus challenge; however, prolonged NLRP3 inflammasome activation induces a hyper-inflammatory state and contributes to pathogenesis and mortality (Thomas et al., 2009; McAuley et al., 2013; Pinar et al., 2016; Tate et al., 2016). Additionally, the expression of NLRP3 was up-regulated in murine brains during influenza viral infection (Yu et al., 2014). Interestingly, the expression of NLRP3 inflammasome-associated genes was also increased in the brains of patients with HIV/AIDS (Walsh et al., 2014). An *in vivo* model of feline immunodeficiency viral infection and HIV-1 Vpr transgenic mice exhibited NLRP3 inflammasome activation with accompanying neuronal loss and neurological disorders (Walsh et al., 2014; Mamik et al., 2016).

### CNS Injury

#### Cerebral Ischemia

The innate immune response plays a critical role in the overall pathogenesis of cerebral ischemia injury. Inflammatory cytokines released from activated microglia initiate downstream signaling cascades that eventually lead to neuronal cell loss following acute brain ischemia (Harari and Liao, 2010). NLRP3 protein was found to increase after experimental ischemic stroke, which was concomitant with high IL-1β and IL-18 levels and extensive neuronal and glial cell death (Lammerding et al., 2016). Interference of NLRP3 activation improved cerebral ischemia outcomes, as evidenced by reduced infarction volumes and decreased levels of neurovascular damage (Fann et al., 2013b; Yang et al., 2014).

#### Traumatic Injury

Traumatic brain injury (TBI) and spinal cord injury (SCI) are both debilitating conditions worldwide and are associated with poor prognosis (Levin and Diaz-Arrastia, 2015; Witiw and Fehlings, 2015). In general, they result from insults by an external mechanical force (Xiong et al., 2013), which is characterized by both primary and secondary injury mechanisms. The primary injury is the immediate mechanical disruption of brain tissue. The secondary injury triggers cascades of cellular and molecular events over a prolonged time course (Werner and Engelhard, 2007; Ji et al., 2012). IL-1β has been widely implicated in the progression of TBI and SCI. The protein levels of the NLRP3 inflammasome components were increased in TBI and SCI patients and in murine models (Adamczak et al., 2012; Liu et al., 2013; Zendedel et al., 2016). Notably, ASC neutralization substantially reduced the contusion volume in a rat model of TBI (de Rivero Vaccari et al., 2009). Pannexin1 channel, in addition to being linked to activation of the NLRP3 inflammasome, serves as a cell death effector during neuronal pyroptosis, which makes it a potential therapeutic target (Adamczak et al., 2014).

#### Hemorrhagic Stroke

Intracerebral hemorrhage (ICH) is a devastating stroke subtype (Keep et al., 2012). The pathophysiology of ICH is characterized by the infiltration of systemic immune cells, the activation of microglia and the production of pro-inflammatory cytokines such as IL-1β (Wang and Doré, 2007). The expression of NLRP3 was increased in a mouse model of ICH, and the inhibition of NLRP3 attenuated neuroinflammation and improved neuronal function, which indicates the involvement of NLRP3 inflammasome in the pathogenesis of ICH (Ma et al., 2014; Yang et al., 2015; Yuan et al., 2015). ROS and the P2X purinergic receptor 7 (P2X7R) pathway may play roles in NLRP3 activation during ICH (Ma et al., 2014; Feng et al., 2015).

Subarachnoid hemorrhage (SAH) is a fatal cerebrovascular disease with the highest mortality among all stroke subtypes (Bian et al., 2012; Keep et al., 2012). Early brain injury, which is highlighted by neuroinflammation, represents a key mechanism of SAH development (Sehba et al., 2012). NLRP3 inflammasome was found to be activated in a rat SAH model (Li J. et al., 2016; Shao et al., 2016). Pharmacological inhibition of P2X7R ameliorated brain edema and neurological deficits, which indicates a mechanism of the P2X7R pathway in NLRP3 activation after SAH (Chen et al., 2013).

### Neurodegenerative Diseases

Neurodegenerative diseases are always accompanied by chronic neuroinflammation with the excessive production of IL-1β and IL-18 pro-inflammatory cytokines, which has detrimental consequences for brain structure and function. The NLRP3 inflammasome is of particular importance in the development of inflammatory responses. To date, a pathogenic role of the NLRP3 inflammasome has been shown in several neurodegenerative diseases including AD, MS and amyotrophic lateral sclerosis (ALS). Whether the NLRP3 inflammasome contributes to the pathogenesis of Prion diseases remains debated.

#### AD

The expression of NLRP3 and caspase-1 is substantially increased in the brains of AD patients (Heneka et al., 2013; Saresella et al., 2016). It has become evident that extracellular deposition of β-amyloid (Aβ) peptides in senile plaques is the initiating event in AD. Aβ drives the release of mature IL-1β via activation of the NLRP3 inflammasome in microglia (Halle et al., 2008; Parajuli et al., 2013). NLRP3 or caspase-1 deficiency substantially attenuates spatial memory impairment and enhances Aβ clearance in AD transgenic mice (Heneka et al., 2013). According to a candidate gene study conducted among northern Han Chinese, two functional single-nucleotide polymorphisms (SNPs) in the *NLRP3* gene (rs2027432 and rs10754558) had synergistic effects on late-onset AD risk (Tan et al., 2013). Moreover, CARD8 protein suppresses NLRP3 activity, and another study indicates that the p.C10X polymorphism of the *CARD8* gene (rs2043211) predisposes people to AD (Fontalba et al., 2008).

#### MS

MS is characterized by the demyelination of axons and chronic inflammation; experimental autoimmune encephalitis (EAE) is the most widely used rodent model for MS. The expression levels of caspase-1, IL-1β and IL-18 were increased in MS plaques and cells from MS patients (Huang et al., 2004; Inoue et al., 2012). NLRP3-deficient mice exhibited resistance to EAE, as evidenced by reduced demyelination and astrogliosis in the spinal cord (Gris et al., 2010). It has been suggested that the NLRP3 inflammasome is likely involved in EAE pathogenesis through the induction of chemokine-mediated recruitment of immune cells (Inoue et al., 2012). However, recent studies have shown that pertussis toxin, which is commonly injected as an adjuvant to increase EAE incidence, induced activation of a pyrin-dependent inflammasome (Dumas et al., 2014; Barclay and Shinohara, 2017), Therefore, the inflammasome sensor involved in MS/EAE might be not restricted to NLRP3.

#### ALS

Mutations in human superoxide dismutase 1 lead to the formation of toxic misfolded protein aggregates, which play an important role in the pathogenesis ALS (Tsuda et al., 1994; Ioannides et al., 2016). Increased NLRP3, ASC, IL-1β, IL-18 and active caspase-1 levels were detected in both human ALS tissue and in murine models (Johann et al., 2015; Debye et al., 2016). Transactive response DNA-binding protein-43 (TDP-43) is considered a major component of intraneuronal aggregates in ALS patients (Arai et al., 2006). TDP-43 instigated NLRP3 inflammasome activation in microglia, which resulted in a pro-inflammatory signaling that is detrimental to motor neurons (Zhao et al., 2015).

#### Prion Diseases

Prion diseases are caused by the conversion of cellular prion protein (PrP^C^) to the pathological isoform PrP^Sc^ through conformational changes (Shi F. et al., 2015). Aggregated PrP^Sc^ peptides lead to the activation of microglia and astrocytes, which results in the release of pro-inflammatory cytokines and neurotoxic factors (Tribouillard-Tanvier et al., 2009). PrP exposure up-regulated NLRP3 and ASC expression in microglia, and silencing of NLRP3 or ASC significantly reduced IL-1β production (Shi F. et al., 2012). However, a recent *in vivo* study using NLRP3- or ASC-deficient mice inoculated with scrapie prions demonstrated that NLRP3 and ASC were not involved in prion pathogenesis (Nuvolone et al., 2015). This discrepancy might be attributed to the different prion proteins used in these studies and the strain-specific features of prion diseases (Tixador et al., 2010; Ayers et al., 2011). More studies on other forms of prion diseases are needed to verify the involvement of the NLRP3 inflammasome.

## Targeting the NLRP3 Inflammasome for the Treatment of Neurological Diseases

**Figure 3:**
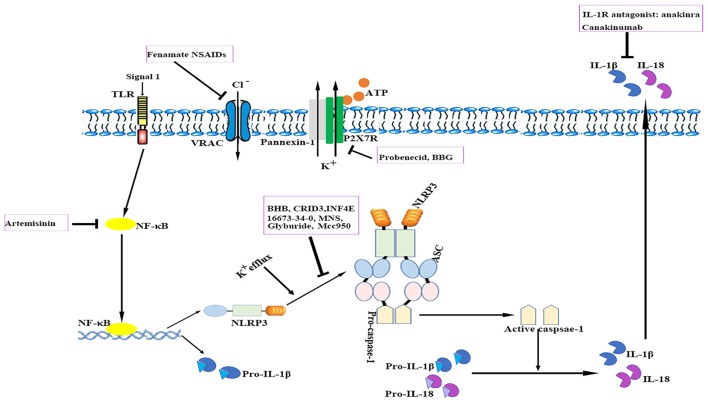
**Therapeutic approaches to targeting the NLRP3 inflammasome.** The cartoon depicts the schematic mode of various therapeutic approaches described in detail in the text. Several steps in NLRP3 activation and the IL-1 pathway have been identified as targets for anti-neuroinflammatory therapies. ASC, apoptosis-related speck-like protein containing a caspase recruitment domain; ATP, adenosine triphosphate; BBG, brilliant blue G; BHB, β-hydroxybutyrate; IL, interleukin; IL-1R, interleukin-1 receptor; MNS, 3,4-methylenedioxy-β-nitrostyrene; NF-κB, nuclear factor-κB; NLRP3, nucleotide-binding oligomerization domain-, leucine-rich repeat- and pyrin domain-containing 3; P2X7R, P2X purinergic receptor 7; TLR, Toll-like receptor.

The relevance of the NLRP3 inflammasome in human CNS pathologies has led to research on the possibilities of pharmacologically targeting the NLRP3 signaling pathways. To date, several advances have been made in the identification of exogenous compounds that may block IL-1 signaling or serve as inhibitors of NLRP3 inflammasome activation (White et al., 2017; see Figure 3); however, most compounds are in the early stages of development.

### Anti-IL-1 Therapy

Currently, anti-IL-1 therapies including interleukin-1 receptor antagonists (IL-1Ra) such as anakinra and specific monoclonal antibodies such as canakinumab have been approved for use in patients with auto-inflammatory disorders (Kuemmerle-Deschner et al., 2011). IL-1Ra administration reduced ischemic brain injury in a murine stroke model; however, it failed to exhibit long-term beneficial effects (Paolicelli et al., 2012). Anakinra is thought to have a preponderance for cryopyrin-associated periodic syndrome (CAPS)-associated neurologic disease because of its better CNS penetration. In addition, several other applications that target IL-1 or IL-1R are also under development (Dinarello et al., 2012). Despite their notable efficacy, anti-IL-1 drugs cannot resolve inflammasome-associated symptoms. Nevertheless, caspase-1-mediated pathways such as pyroptosis also drive disease pathology. These findings suggest that the direct blockade of inflammasome activation instead of merely neutralizing its downstream cytokines may be advantageous for controlling unwanted inflammatory reactions. Moreover, anti-IL-1 therapies are expensive and most of them may not readily penetrate tissues such as the brain.

### Small Molecule Inhibitors of NLRP3

Compounds with a sulfonylurea moiety appear to specifically inhibit activation of the NLRP3 inflammasome in the activation stage without affecting the NF-κB signaling-dependent priming stage (Lamkanfi et al., 2009; Coll et al., 2015). Glyburide was the first identified sulfonylurea moiety-containing drug to exhibit NLRP3-inhibitory activity *in vitro*; however, the required *in vivo* dose is associated with hypoglycemic effects. MCC950, a small-molecule compound that shares similarities with sulfonylurea, was shown to block ASC oligomerization induced by NLRP3, which makes it a highly potent and selective NLRP3 inhibitor. It effectively attenuated the inflammatory response in murine EAE models and *ex vivo* human samples (Coll et al., 2015). Its role in other neurological diseases requires additional investigation. Additionally, 16673-34-0, an intermediate substrate in glyburide synthesis, exhibited no effect on glucose metabolism and has been demonstrated to ameliorate myocardial ischemia/reperfusion (I/R) injury by inhibiting the formation of the NLRP3 inflammasome (Marchetti et al., 2014).

The ketone metabolite β-hydroxybutyrate (BHB) was discovered to suppress NLRP3 inflammasome activation by inhibiting NLRP3-ASC oligomerization (Youm et al., 2015). Experiments showed that BHB decreased K^+^ efflux and endoplasmic reticulum stress (Youm et al., 2015; Bae et al., 2016). Furthermore, BHB is transported to brain parenchyma and plays a neuroprotective role under several pathological conditions (Orhan et al., 2015; Xie et al., 2015).

A cysteinyl leukotriene receptor antagonist, which had been initially incorrectly termed as CRID3, was found to prevent caspase-1 activation in response to NLRP3 activators via direct inhibition of ASC oligomerization (Coll et al., 2011, 2015).

INF4E is a newly synthesized compound that directly inhibits NLRP3 ATPase and specifically suppresses NLRP3 inflammasome activation (Cocco et al., 2014). It was shown to be protective against NLRP3-involved myocardial I/R in a rat model (Mastrocola et al., 2016). Further rigorous investigations are needed to evaluate the effects of INF4E on neurological diseases and its side effects.

3,4-methylenedioxy-β-nitrostyrene (MNS), a novel tyrosine kinase inhibitor, was reported to specifically and potently inhibit NLRP3 by directly targeting its NOD and LRR domain (He and Amer, 2014). According to a recent study, MNS prevented wound progression and improved healing in an experimental burn model (Xiao et al., 2016). The potent effects and minimal cytotoxicity of MNS make it an attractive candidate for the treatment of neurological diseases; however, studies investigating the role of MNS in the CNS are lacking (Hsieh et al., 2010).

### Other Compounds that Target Specific Pathways

Artemisinin, a well-established antimalarial drug, has been verified to exert anti-inflammatory effects via inhibition of the NF-κB signaling pathway. Artemisinin treatment also reduced the neuritic plaque burden and NLRP3 inflammasome activity in an AD transgenic mouse model (Shi et al., 2013). Studies have shown that artemisinin induces various side effects such as neurotoxicity, cardiotoxicity, embryotoxicity and allergic reactions when administered long-term (Efferth and Kaina, 2010; Li and Hickman, 2011).

The ATP-gated receptor P2X7R has been implicated in activation of the NLRP3 inflammasome (Deplano et al., 2013). P2X7R antagonist brilliant blue G (BBG) alleviated inflammation and improved neurological functions in a rodent model of SAH (Chen et al., 2013). BBG can penetrate the BBB at relatively low doses (Diaz-Hernandez et al., 2012). However, the use of P2X7R antagonists is controversial as these receptors are located in various cell types under pathological conditions and they may induce undesirable off-target effects (Franke et al., 2004).

Probenecid, an FDA-approved drug for gout and hyperuricemia treatment, is a specific Pannexin1 channel blocker (Silverman et al., 2009). Probenecid inhibited NLRP3 activation in cultured neurons and astrocytes by providing a high extracellular concentration of K^+^ and decreased caspase-1 expression in the brains of aged rats (Mawhinney et al., 2011; Jian et al., 2016).

A recent study reported that fenamate non-steroidal anti-inflammatory drugs (NSAIDs), including flufenamic acid and mefenamic acid, were neuroprotective in rodent models of AD (Daniels et al., 2016). Fenamates selectively inhibited NLRP3 by blocking volume-regulated anion channels (VRAC) in macrophages (Daniels et al., 2016). Fenamate NSAIDs target both VRAC/NLRP3 and cyclooxygenases, which makes them more efficacious than those targeting a single point in one inflammatory pathway. However, fenamate NSAIDs are associated with CNS toxicity, gastrointestinal adverse effects, nephrotoxicity, metabolic acidosis and prolongation of prothrombin time (Kingswell, 1981; Redmond, 1981; Frank et al., 1983; Court and Volans, 1984; Kamour et al., 2016). It is important to reassess the benefit-risk profile of fenamate NSAIDs for treating NLRP3-associated disorders.

## Outstanding Questions

To date, several hurdles that require further investigation remain. Because the mechanisms of NLRP3 activation are differentially regulated at the cell and tissue levels (Toldo et al., 2015), it is necessary to characterize each step of the NLRP3 inflammasome cascades in specific cell types and brain regions under different pathological contexts. Additionally, the potential roles of NLRP3 signaling in the regulation of specific cell interactions that are involved in neuroinflammation remain to be elucidated.

## Conclusions and Future Directions

Major advances in the understanding of inflammasomes and inflammasome-mediated disorders have been made in the past decade. A spectrum of inflammatory responses has been associated with CNS pathological circumstances; thus, inflammasome activation likely exerts strong influences on various neurological diseases. Insufficient activation of inflammasome causes the host to become vulnerable to PAMPs and DAMPs; nevertheless, excessive inflammasome activation causes unfavorable outcomes in diverse diseases (Rathinam et al., 2012). Thus, the manipulation of a balanced and effective inflammasome-mediated inflammatory response is of paramount importance.

Recent years, Cre-lox recombination-mediated neurogenetics has been developed as a useful technique to generate cell-specific gene knock-outs (KO) and knock-ins (Witten et al., 2011). Conditional autophagy gene KO mice have been recently generated by breeding autophagy-deficient mice with specific Cre drivers to investigate the regulatory role of autophagy in NLRP3 inflammasome activation in microglia (Cho et al., 2014). It is plausible to generate microglia/astrocyte conditional NLRP3 KO and knock-in mice through a similar genetic approach to study cell-specific interactions.

## Author Contributions

LS and LP made equal contribution to this work, performed literature review and drafted the article. SY revised and critically appraised the manuscript for intellectual content. YS and YW contributed to the revision and edition of the manuscript. All authors read and approved the final manuscript.

## Conflict of Interest Statement

The authors declare that the research was conducted in the absence of any commercial or financial relationships that could be construed as a potential conflict of interest.
